# Supplementary material for: Reconciling Mining with the Conservation of Cave Biodiversity: A Quantitative Baseline to Help Establish Conservation Priorities
Source: PLoS One. 2016 Dec 20;11(12):e0168348. doi: 10.1371/journal.pone.0168348 (PMC5173368; doi:10.1371/journal.pone.0168348)
Supplement: S1 Dataset — (ZIP) [file pone.0168348.s002.zip › Taxa/Serra Norte/SN_2013/N5S/Lista N5S-82.pdf]

CAVIDADE N5S-0082

Classe	Ordem	Fam/Outros	Gên/Outros	Espécie	S-U	U-S	Zon.
Arachnida	Amblypygi	Phrynidae	*Heterophrynus*	*longicornis*		1	E
Arachnida	Araneae	Araneidae		jovem	X	X	E
Arachnida	Araneae	Ochyroceratidae		jovem		X	E
Arachnida	Araneae	Pholcidae	*Mesabolivar*	*aurantiacus*	X	X	E
Arachnida	Araneae	Salticidae		jovem		X	E
Arachnida	Araneae	Tetragnathidae		jovem	X		E
Arachnida	Araneae	Theridiidae	*Theridion*	sp.2	X		E
Arachnida	Araneae	Theridiosomatidae	*Plato*	sp.1		X	E
Arachnida	Opiliones	Escadabiidae		sp.1		X	E
Chilopoda	Scutigeromorpha	Pselliodidae	*Sphendononema*	*guildingii*		1	E
Diplopoda	Polydesmida	Chelodesmidae		jovem		1	E
Diplopoda	Polydesmida	Pyrgodesmidae		jovem		X	E
Entognatha	Collembola	Entomobryidae		sp.4	X		E
Entognatha	Collembola	Paronellidae		sp.1		X	E
Insecta	Coleoptera	Staphylinidae	Pselaphinae	sp.1		X	E
Insecta	Diptera	Cecidomyiidae	Cecidomyiinae	sp.	X		E
Insecta	Diptera	Simuliidae		sp.		X	E
Insecta	Diptera	Tipulidae	Tipulinae	sp.	X	X	E
Insecta	Homoptera	Cixiidae		sp.4	X		E
Insecta	Hymenoptera	Formicidae	*Camponotus*	sp.1	X		E
Insecta	Hymenoptera	Formicidae	*Camponotus*	sp.2	X		E
Insecta	Hymenoptera	Formicidae	*Crematogaster*	sp.1	X		E
Insecta	Hymenoptera	Formicidae	*Pachycondyla*	sp.2		X	E
Insecta	Hymenoptera	Formicidae	*Paratrechina*	sp.1	X		E
Insecta	Isoptera			sp.	X		E
Insecta	Lepidoptera	Noctuoidea		sp.3		2	E
Insecta	Orthoptera	Phalangopsidae	*Paraclodes*	jovem	2	1	E
Insecta	Psocoptera			jovem	X	X	E
Insecta	Thysanura	Ateluridae		jovem		X	E
Gastropoda	Pulmonata	Systrophiidae	*Happia*	sp.		X	E
